# What Does It Take for an Infant to Learn How to Use a Tool by Observation?

**DOI:** 10.3389/fpsyg.2016.00267

**Published:** 2016-03-01

**Authors:** Jacqueline Fagard, Lauriane Rat-Fischer, Rana Esseily, Eszter Somogyi, J. K. O’Regan

**Affiliations:** ^1^Laboratoire Psychologie de la Perception, UMR 8242, CNRS – Université Paris DescartesParis, France; ^2^Department of Zoology, University of OxfordOxford, UK; ^3^Laboratoire Éthologie, Cognition, Développement, Université Paris-Ouest-Nanterre-La-DéfenseNanterre, France

**Keywords:** observational learning, demonstration, tool use, social cues, infants

## Abstract

Observational learning is probably one of the most powerful factors determining progress during child development. When learning a new skill, infants rely on their own exploration; but they also frequently benefit from an adult’s verbal support or from demonstration by an adult modeling the action. At what age and under what conditions does adult demonstration really help the infant to learn a novel behavior? In this review, we summarize recently published work we have conducted on the acquisition of tool use during the second year of life. In particular, we consider under what conditions and to what extent seeing a demonstration from an adult advances an infant’s understanding of how to use a tool to obtain an out-of-reach object. Our results show that classic demonstration starts being helpful at 18 months of age. When adults explicitly show their intention prior to demonstration, even 16-month-old infants learn from the demonstration. On the other hand, providing an explicit demonstration (“look at how I do it”) is not very useful before infants are ready to succeed by themselves anyway. In contrast, repeated observations of the required action in a social context, without explicit reference to this action, considerably advances the age of success and the usefulness of providing a demonstration. We also show that the effect of demonstration can be enhanced if the demonstration makes the baby laugh. Taken together, the results from this series of studies on observational learning of tool use in infants suggest, first, that when observing a demonstration, infants do not know what to pay attention to: demonstration must be accompanied by rich social cues to be effective; second, infants’ attention is inhibited rather than enhanced by an explicit demand of “look at what I do”; and finally a humorous situation considerably helps infants understand the demonstration.

## Introduction

Infants are avid explorers of the environment: their intrinsic motivation drives them to constantly look for new experiences which, in turn, increases their knowledge of the environment and allows them ultimately to display typically human behaviors such as tool use. In particular, it has been hypothesized that the “origins of tool use in humans can be found […] in the perception-action routines that infants repeatedly display as they explore their environments." (Lockman, 2000, p. 137). But whereas such a mechanism of discovery is undoubtedly an important factor in development, another, more economical, but less studied mechanism also exists, namely observational learning.

Observational learning can be defined as the process whereby an adult or a child “attempts to imitate another person executing a new motor skill” (Hayes et al., 2008, p. 407). Imitation is a rapid and efficient means to learn a new skill, allowing the learner to avoid painstaking trial-and-error learning. Whereas some imitation can be observed from birth, for instance for mouth opening, and whereas as early as 6 months infants can repeat the manual action an adult makes in front of him, such as squeezing a duck (Abravanel et al., 1976), true observational learning appears much later, not before the second year (Meltzoff, 1988; Elsner and Aschersleben, 2003; Elsner et al., 2007; Esseily et al., 2010).

In this paper, after a short reminder of what is known about observational learning during early development, the conditions leading to successful imitation, and after briefly presenting the tool-use problem and the spontaneous behavior of 12–22 month-old infants confronted with this problem, we will review studies we have been performing in our laboratory which investigate possible reasons for the late appearance of observational learning in our tool-use task. One issue we will consider is whether it is possible to advance the age of observational learning and in which conditions of context and of demonstration.

## Observational Learning

As said above, observational learning is a special case of imitation, in which the action to be imitated is not part of the child’s existing repertoire of actions or which is failed without prior demonstration. Thus, whereas imitation of simple actions can be observed as early as 6 months of age (for reviews see Poulson et al., 1989; Elsner, 2007; Elsner et al., 2007), this cannot be considered to be observational learning, because these simple actions are already in the infant’s motor repertoire. Observational learning of a new skill has been less studied, at least in infants and toddlers (see Ashford et al., 2007’s meta-analysis for children and adult studies). In one study it was shown that at 12 months, infants can learn by observation how to bimanually manipulate a rolling drum to produce music (Fagard and Lockman, 2009). At 14–15 months they can learn by observation to push a button to produce music (Meltzoff, 1988; Elsner and Aschersleben, 2003). In the latter study they are surprised if the effect they produce is not similar to that produced by the adult. In another study it appeared that at 15 months, children can learn by observation how to turn a bottle upside down to retrieve a small peg inserted in it (Esseily et al., 2010).

Observational learning seems to occur later for tool use. For instance, Chen and Siegler (2000) showed that even well after 18 months, infants may still be unable to learn how to use a tool through observation. Between 18 and 35 months, some infants used the tool appropriately to retrieve a toy after observation, but others still used indirect strategies such as trying to reach with their hands, asking for their mother’s help or simply staring at the toy without trying to reach for it. In the pilot testing of their Nagell et al. (1993) study, also noticed that the three 18-month-olds they observed were unable to use a rake to get a toy out of a cage after demonstration from the experimenter. In contrast, in the 1993 study itself, some 2-year-olds showed occasional successes, more so in the groups which had been shown the action first (2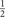 successes out of 10) than in the no-model group (less than 1/10 success). Thus, learning a complex multiple-step skill by observing an adult is difficult before the end of the second year of life. Before considering tool use as part of the larger category of two-step or “means-end” actions acquired during early childhood, we will briefly review the studies in which manipulating the modeling conditions impacted on infants’ success in reproducing the modeled action.

## Factors Influencing the Reproduction of Modeled Actions in Young Children

Many studies have been devoted to understanding how infants and young children reproduce the action modeled by an adult. It has been traditionally said that, as opposed to non-human primates, young infants tend to imitate not only the goal of the demonstrated action (emulation) but also the means used by the model to reach this goal (Nagell et al., 1993), sometimes even over-imitating irrelevant means (Whiten et al., 2009; McGuigan and Robertson, 2015). A growing body of research has tried to understand the factors leading infants to either imitate the means or else to only emulate the goal.

These studies have revealed several factors. Young children predominantly imitate the means when the set-up makes the goal of the action less clear (e.g., reaching toward the table without (vs. with) a dot marking the point of reaching, (Bekkering et al., 2000; see also Carpenter et al., 2002, 2005; Williamson and Markman, 2006). On the other hand, infants tend to emulate the goal when the model shows an irrational means (e.g., pushing a toy through a tube using a stick toward the free end as opposed to toward the dead-end, Want and Harris, 2001); when the model uses a means for a clear reason but that is not applicable to themselves (e.g., switching on a light with the head, with the model’s hands being occupied, Meltzoff, 1988; Gergely et al., 2002; Zmyj et al., 2009); when the means used successfully by the model seems accidental (“Whoops”) rather than intentional (“There”) (Carpenter et al., 1998); when the information available from the model is degraded (e.g., obtaining a reward from a box following a video rather than a live model, McGuigan et al., 2007). In addition, young children are more successful in their imitation when the demonstration comes after the intention of the model is shown (Carpenter et al., 2002; Southgate et al., 2009).

The above studies, many of them involving children older than 2 years, show the importance of taking into account different factors that can influence the child’s reproduction of an action modeled by an adult. However, in contrast with our task, many of the actions to be repeated by the child were simple and probably familiar to the child (with a few exceptions: Carpenter et al., 1998; Want and Harris, 2001; McGuigan et al., 2007). In our original study, the task was difficult and infants did not spontaneously succeed at retrieving an out-of-reach toy with a rake placed within reach but not next to the toy. We thought that the goal of the action was clear to the infant since the demonstration always took place after the infant had tried unsuccessfully to retrieve the toy (we later questioned this assumption, as we will see further). The means used by the model was always direct (the model grasped the rake and raked the toy toward himself or herself). Finally, in our task there was no other way to succeed than the one shown by the adult. Before discussing the factors that could explain why infants failed to copy the demonstration in our task before 18 months of age, we will briefly review what is known about the development of means-end actions of which tool use is a special example.

## From Means-End to Tool Use

A means-end task is a task in which the goal of the action cannot be manually reached directly by the actor, who has to perform intermediate actions or “means” (Piaget, 1936). One of the earliest cases when infants are confronted with the impossibility of directly grasping an interesting object is when the interesting part of the object is at the end of a handle, too far away to be grasped directly (e.g., a rattle). For instance we observed that when a bright ball is at the end of an uninteresting rigid handle, 6-month-old infants point toward the ball while ignoring the handle. In contrast, most 8-month- and 10-month-old infants immediately grasp the handle while looking at the ball (Fagard et al., 2015).

When the uninteresting part is not rigid, so that the composite object looks like two objects rather than a single one, for instance a toy at the end of a string or placed on a cloth, it takes a few more weeks for the child to understand that she or he can pull the string or the cloth to retrieve the object of interest (Piaget, 1936; Fagard, 1998; Willatts, 1999; Buttelmann et al., 2008).

Bates et al. (1980) compared 9–10-month-old infants retrieving an out-of-reach toy placed either on a cloth, at the end of a string, or at different positions near three kinds of utensils likely to help the children retrieve it (hoop, crook, or stick). The children succeeded in conditions where toy and means to retrieve it were physically linked (“unbreakable contact,” cf. means-end situations just mentioned) but less often when the contact was breakable, and not at all in the condition with no contact. The authors concluded that at 10 months, solving the problem is easier when the spatial arrangement suggests a link between the means to retrieve it and the toy.

A generally accepted definition of tool use is the ability to use one object to extend the limit of our physical body in order to act upon another spatially independent object (Beck, 1980). Infants’ first successful use of such a real tool is likely to be with a spoon, starting around the age of 1 year. This skill progresses considerably during the second year (Connolly and Dalgleish, 1989). Note that the case of the spoon is particular, in the sense that prior to using the spoon themselves, infants have many opportunities to see their family and other people use a spoon to eat.

Using unfamiliar tools to bring an out-of-reach object within reach is succeeded later. A few studies have focused on how infants learn to use such a new tool (see Greif and Needham, 2011 and Keen, 2011, for reviews). Most of them have focused on perceptual factors, all stressing that difficulty increases with the size of the spatial gap between the tool and the object to be acted upon (Bates et al., 1980; van Leeuwen et al., 1994), and more generally with the number of steps needed to achieve the required result (Smitsman and Cox, 2008). In these studies, emphasis was put either on the sensorimotor progress leading to skillful tool use (Connolly and Dalgleish, 1989), on the perceptual constraints which make using a tool a real cognitive problem for the infant (Bates et al., 1980; van Leeuwen et al., 1994; Smitsman and Cox, 2008), or on the role of familiarity or novelty in the capacity to use a tool or to transfer and generalize knowledge to new tools (Brown, 1990; Barrett et al., 2007). In other words, most of these early studies were concerned more with cataloging the factors inducing success than with understanding the actual mechanisms underlying tool-use learning, in particular trial and error and observational learning.

In the following section we recall data from a series of recently published studies on the emergence of tool use (Rat-Fischer et al., 2012; Fagard et al., 2014), in which we investigated to what extent trial and error and observational learning respectively allow infants to learn how to use a tool. Here we aim at summarizing the parts that concern observational learning. Thus, we will only briefly recall the methodology, referring the reader to the original articles for further details.

## Using a Tool to Bring a Far-Away Toy into Reach

**FIGURE 1:**
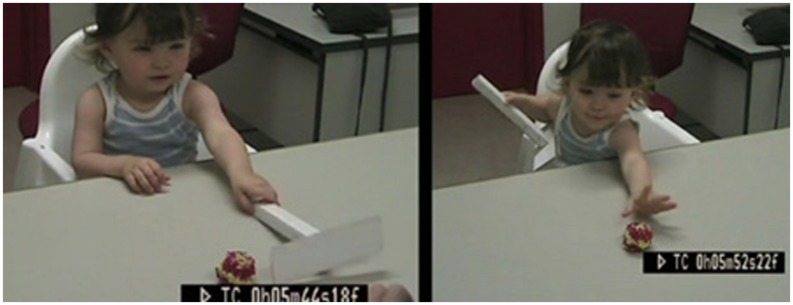
**Typical spontaneous behavior of a 16-month-old when an out-of-reach toy is presented to the side of a rake (spatial gap): the child grasps the rake, discards it, and begs for the toy**.

Our paradigm consisted of presenting infants with a desirable out-of-reach toy, and with a T-shaped rake-like tool, long enough to retrieve the toy. The “rake” was within reach and constructed out of white cardboard with a 20-cm-long handle (Fagard et al., 2014). The toys were small, bright, and salient whereas the rake was white and intentionally unobtrusive so that infants would be attracted to the out-of-reach toy more than to the rake. We investigated several spatial arrangements of toy and rake (toy inside/against the rake, toy inside the rake but not against, toy to the side of the rake). We followed five infants from 12 to 20 months in a longitudinal study (Fagard et al., 2014) and 60 infants aged 14, 16, 18, 20, 22 months, in a cross-sectional study (Rat-Fischer et al., 2012). Here only the condition “toy to the side of the rake” will be considered (see **Figure 1**) since it was only in this condition that we investigated the effect of demonstration in further experiments. For more details, see Rat-Fischer et al. (2012) and Fagard et al. (2014).

In the longitudinal study, all infants younger than 16 months failed to retrieve the toy, except for one isolated success that the infant could not repeat. There was a sudden increase of spontaneous success between age 17 months and age 18 months. At around 18 months, all five infants succeeded at least at some trials. In the cross-sectional study, where each child had only one session, there was 0% spontaneous success at 14 and 16 months, and the percentage slowly increased to reach 10% at 18 months and about 30% at 22 months.

Thus, spontaneous success at tool use when the tool and the out-of-reach object are spatially separated developed slowly during the second year, though faster in the longitudinal study when the infants were given the test every month from their first birthday. These results were not surprising in light of earlier studies showing that a spatial gap between tool and object renders the task extremely difficult for children less than 2 years (Bates et al., 1980; Brown, 1990; van Leeuwen et al., 1994). What we expected less is that demonstration by an adult did not increase the rate of success before 18 months, as we will see in the next section. Since observational learning of means-end has been shown possible as of 12 months of age (Meltzoff, 1988; Elsner and Aschersleben, 2003; Elsner et al., 2007), and since infants are able to use a spoon at about the same age (Connolly and Dalgleish, 1989), we expected the demonstration in our situation to be useful before 18 months of age.

## Classic Demonstration of a Tool-Use Action

**FIGURE 2:**
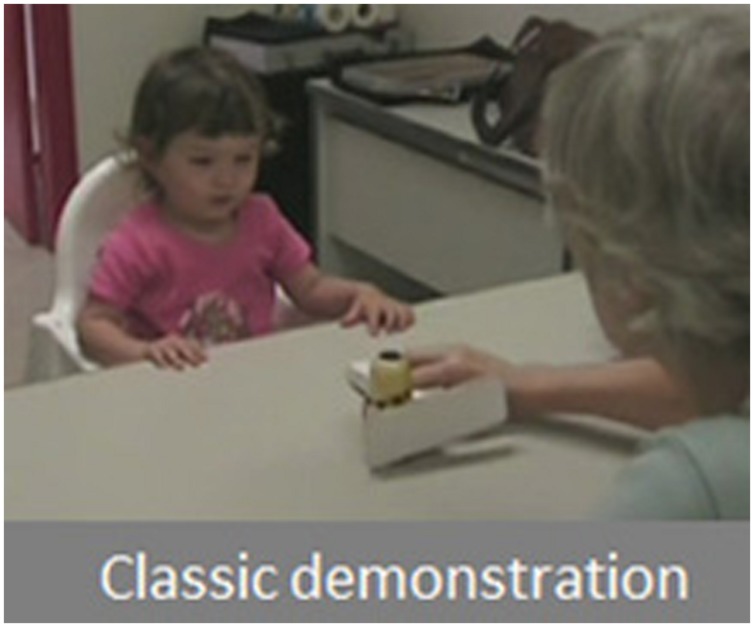
**Classic demonstration**.

In both the longitudinal and the cross-sectional studies, each time infants failed to retrieve the toy, an adult (the experimenter or the parent) gave two consecutive demonstrations, from the infant’s point of view (i.e., the experimenter or parent moved the rake and toy toward the child, see **Figure 2**). Then the infants were tested again.

In the longitudinal study, none of the infants succeeded in retrieving the toy with the rake immediately after demonstration before the age of 18 months.

**FIGURE 3:**
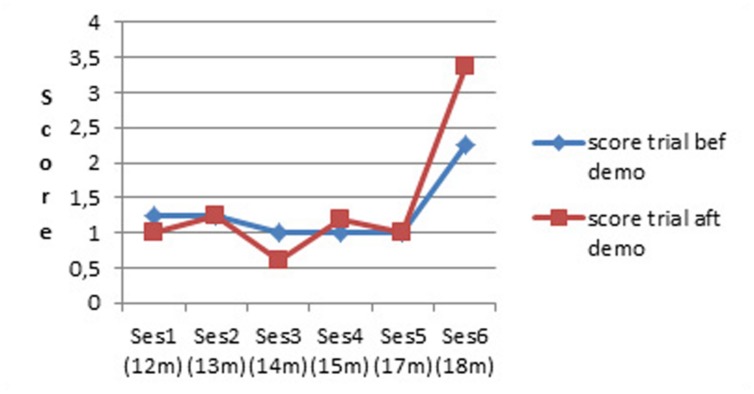
**Compared score before and after a classic demonstration (longitudinal study)**.

To check whether the demonstration had some effect even though infants were not actually able to retrieve the toy, we defined a performance score between 0 and 4 as follows. 0: no interest neither in the toy nor in the rake; 1: mostly interested in the out-of-reach toy, pointing toward it and trying to retrieve it without using the rake; 2: mainly interested in manipulating the rake; 3: repeatedly bringing the rake to bear on the toy but seemingly not with the purpose of retrieving the toy; 4: successful or near successful retrieval of the toy with the rake. There was no difference in score before versus after demonstration during the first five sessions (up to age 17 months). Only at the sixth session (age 18 months) did the statistics show that infants scored significantly higher after the demonstration (see **Figure 3**). We found similar results in the cross-sectional study (Rat-Fischer et al., 2012). Thus infants started to benefit from demonstration quite late, not before 18 months.

The relatively late effect of demonstration is consistent with other work showing that proper understanding of the causal structure of means-end tasks in observational learning only matures in the second half of the second year (Meltzoff, 1995; Bellagamba and Tomasello, 1999; Huang et al., 2002). However, our observation of the absence of an effect of demonstration before 18 months contrasts with the studies mentioned above showing that infants can learn to solve a means-end task from observation of a demonstration by an adult from the beginning of their second year of life (Provasi et al., 2001; Elsner et al., 2007; Esseily et al., 2010). This led us to investigate the reasons why repeated demonstrations were not effective in our studies, in other words, what are the factors that could explain why infants failed to copy the demonstration in our task? The first possibility we investigated was that infants were not able to interpret the demonstration because they did not sufficiently understand the intention of the demonstrator. To test this possibility, we provided the infant with cues about the demonstrator’s intention prior to demonstration.

## Showing the Observer’S Intention Prior to Demonstration

**FIGURE 4:**
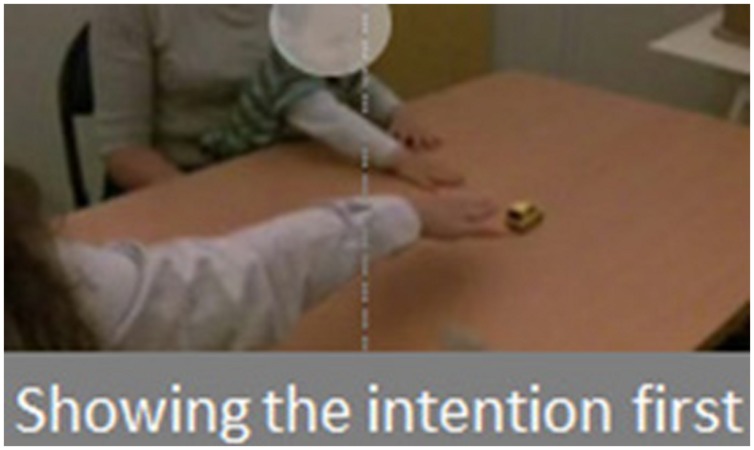
**Showing the intention prior to demonstration**.

In this study (Esseily et al., 2013), we tested 70 16-month-old infants for tool use. The toy consisted of a small car which could be rolled along the table. We used the same rake as for the longitudinal and cross-sectional studies described above, and presented infants with a condition with a spatial gap between rake and toy. We chose age 16 months because we knew from the two previous studies that at this age infants could not spontaneously succeed at this task when the toy is not contiguous with the rake. We nevertheless used a control group with no demonstration (spontaneous group), which we could compare with two demonstration groups and two other control groups (*N* = 14 in each group). For the demonstration groups, the experimenter sat perpendicular to the infant and received the car from another person seated in front of the child. The experimenter played with the car for a few seconds, and then rolled it along the table in front of her so that it ended up out of reach, but within reach of the rake. Then, depending on the group it belonged to, each infant either received a classic demonstration (the experimenter simply grasped the rake and used it to retrieve the car), or the infant was shown the intention of the experimenter before demonstration. To do this, once the toy was out of reach, the experimenter stretched her arm and hand toward the car, obviously trying to grasp it and said, “I can’t get it” (see **Figure 4**). She then used the tool to retrieve the car. In both conditions the same scenario was repeated twice (for more details about the protocol, see Esseily et al., 2013). After demonstration, infants received the same test as was given directly at the beginning of the session to the infants of the spontaneous group. To make sure that a difference between the classic and the prior intention demonstrations could not be due to the fact that the attention of the infant was enhanced on the car, rather than to understanding of the experimenter’s intention, we added another control group (Stimulus enhancement condition). In this condition, once the car had been rolled out of reach by the experimenter, the person seated across from the infant made the car move by itself for a few seconds by manipulating a magnet under the table. The experimenter then performed the demonstration as in the Classic demonstration condition, followed by the test. And to make sure that a difference between the classic and the prior intention demonstration could not be due to more “motor resonance” (Paulus et al., 2011) when the experimenter showed her intention (since here the arm movement toward the car is repeated twice), we added a further condition where the experimenter stretched her arm toward the empty place where the car was located in the demonstration condition (Motor resonance condition). The experimenter then performed the demonstration as in the Classic demonstration condition, followed by the test.

**FIGURE 5:**
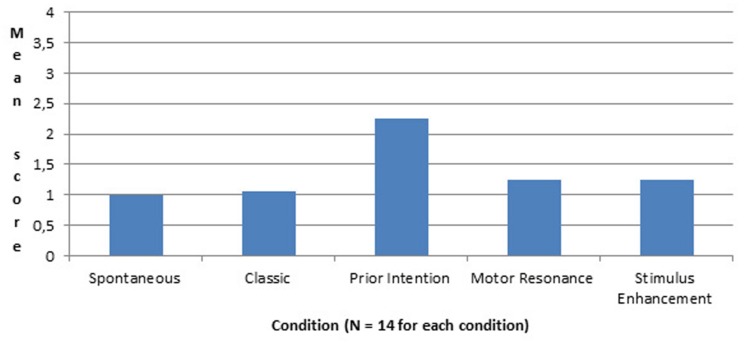
**Mean score at the test following demonstration (except for the Spontaneous group) as a function of condition**.

We compared infants’ scores for the first action at the test, the best action and the mean score for all actions. We found that there was a significant effect of condition, due to the difference between the prior intention group and all other groups. Infants used the rake in connection with the toy significantly more often after watching the experimenter showing her intention prior to demonstration than after a classic demonstration. And this effect is unlikely to be due to stimulus enhancement or to motor resonance (see **Figure 5**).

This result suggests that one of the reasons infants do not learn from observing a demonstration of unfamiliar tool use is that they do not understand the intention of the experimenter when he or she does the classic demonstration. This is consistent either with the teleological reasoning theory suggesting that infants need to understand the intended goal of the experimenter in order to understand her actions and selectively imitate them (Gergely and Csibra, 2003), or with a more mentalistic view (Buttelmann et al., 2008). It is in accordance with the studies showing more successful imitation of the means when the demonstration comes after the intention of the model is shown (Carpenter et al., 2002; Southgate et al., 2009).

## Implicit Repeated Demonstration of Tool Use

Another possibility to explain why in previous studies we failed to show observational learning may be that providing a few demonstrations in a single session is not an effective way to learn. In real life, infants have ample opportunity over many months to observe adults around them using tools. For example, as already mentioned, infants start understanding the affordance of a spoon after many opportunities to watch the functionality of the spoon when seeing people around them eat with a spoon. In addition, parents’ or caregivers’ demonstrations are implicit rather than explicit as in the demonstrations used in our studies. Parents rarely tell their children “look how I hold my spoon to eat”! We decided to investigate what would happen if infants had a similar opportunity, over an extended period of time, to watch an adult repeatedly use a rake to move objects. We opposed this condition with a condition where infants had the opportunity to manipulate a tool in the presence of toys, all within reach, without any demonstration from the adults. Thus we contrasted the effect of implicit repeated demonstration without practice with that of practice manipulation without demonstration.

In this study Somogyi et al. (2015), 18 infants were followed from the age of 14 months over 6 weeks. We used the same rake as for the previous studies, and toy and rake were presented with a spatial gap between them. We knew from the previous studies that in this age range, and with a spatial gap, infants would not spontaneously succeed. We compared the performance at 16 months depending on the kind of familiarization received with the tool. In one group (Visual familiarization, 10 infants), the infants observed an adult use a rake to bring a toy toward the infant, doing so without any verbal comment so as to avoid explicit teaching. The action was repeated eight times, each time with a different toy. The infants were never given the rake during this familiarization phase.

In the other group (Manual familiarization, 8 infants), the rake was placed on the table near the infant, next to a few toys, and the infants were allowed 5 min to freely interact with the rake and the toys. No instruction or demonstration was given. We decided to use this manual control group in response to colleagues’ suggestions that perhaps infants do not learn by observation because they are not manually familiar with the tool and thus the action is motorically too demanding, making it difficult for the infant to pick up the relevant information during demonstration.

All infants came to the lab for the first session: they were first tested on the spontaneous use of the rake as in the condition of spatial gap described above, to confirm that they all spontaneously failed at the task. They were then assigned to one of the two groups. All infants of the Visual group received Visual familiarization from the experimenter and all infants of the Manual group received Manual familiarization as described above. For both kinds of familiarization the parent present in the lab was taught the procedure he or she would have to use once a week at home with the infant for the following 5 weeks. In addition, one of the experimenters went to visit all the families every other week so as to check that the familiarization had been well understood by the parent. All infants came to the lab for the seventh session: they were first tested on the spontaneous use, and if they failed they were given two classic demonstrations from the experimenter followed by a test.

We observed a significant effect of test time and a significant interaction between test time and familiarization condition, indicating a significant effect of familiarization in the case of Visual familiarization, but not in the case of Manual familiarization.

**FIGURE 6:**
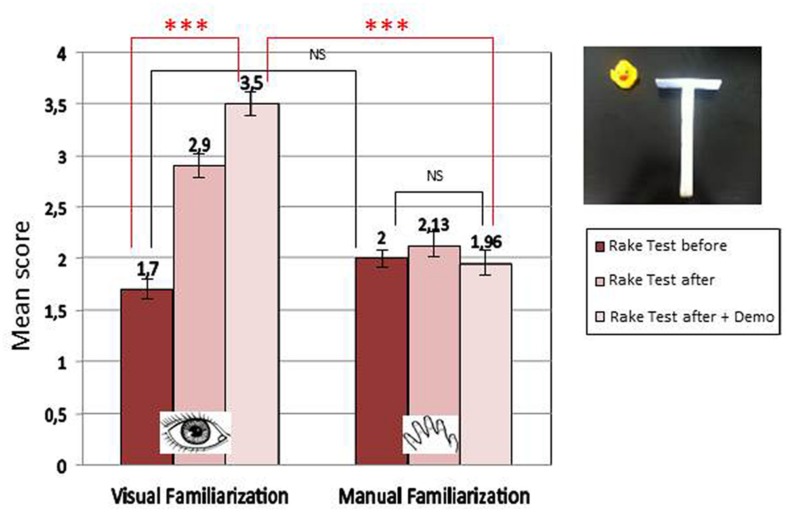
**Mean score before and after familiarization as a function of time and condition of familiarization (^∗∗∗^*p* < 0.0001)**.

We also compared the highest score obtained at the seventh session before and after demonstration, as a function of the kind of familiarization. Again we found a significant interaction, with a significant effect of familiarization in the case of Visual familiarization, but not in the case of Manual familiarization. This shows that infants of the Visual familiarization group increased their performance significantly more after demonstration than infants of the Manual familiarization group (see **Figure 6**).

The interesting point raised by these results is that they show that infants can improve their performance at tool use without manual familiarization with the tool. Repeated purely visual demonstrations of the functionality of the tool, made in a natural way, without explicit reference to its use, is enough to significantly advance the age of successful tool use.

These results first show that it is not the lack of manual skill which leads to the absence of learning from classic demonstration. They also suggest that infants may need several demonstrations over an extended time period to learn by observation. Most importantly, our results suggest that implicit demonstration in an ecological setting may be more efficient than explicit teaching.

## Making the Infant Laugh During Demonstration

Evidence from an additional study we performed suggest another, less studied, factor influencing observational learning, namely emotional state. In a pilot study where we were pre-testing ways of giving the demonstration, we observed with surprise that, when occasionally infants were amused by our demonstration and laughed, these laughing babies would imitate us immediately after a demonstration. They did this with a level of skill that we had never observed after other demonstrations. We thus decided to experimentally test the effect of laughing on the ability to learn from a demonstration.

In this study (Esseily et al., 2015), we tested 51 18-month-old infants. We chose this age because it is the youngest age when classic demonstration of tool use starts to be effective. We eliminated 11 infants who succeeded spontaneously at the first tool-use test given before the demonstrations started. We used the same rake as for the previous studies and toy and rake were presented with a spatial gap between them. Among the 40 infants who failed spontaneously at the first test and thus were kept in the study, 10 infants received a classic demonstration, and the other 30 infants received a humorous demonstration. In the humorous demonstration, the experimenter took the rake with one hand, used it to bring the toy closer, then reached for the toy with the other hand and threw it onto the floor immediately. Since only one third of the infants laughed after such a demonstration, we ended up with three groups, Classic demonstration group (10 infants), Humorous demonstration/infants not laughing group (20 infants), Humorous demonstration/infants laughing group (10 infants). In all three groups the infants were tested before and after eight demonstrations, which varied according to the group the infant was assigned to.

**FIGURE 7:**
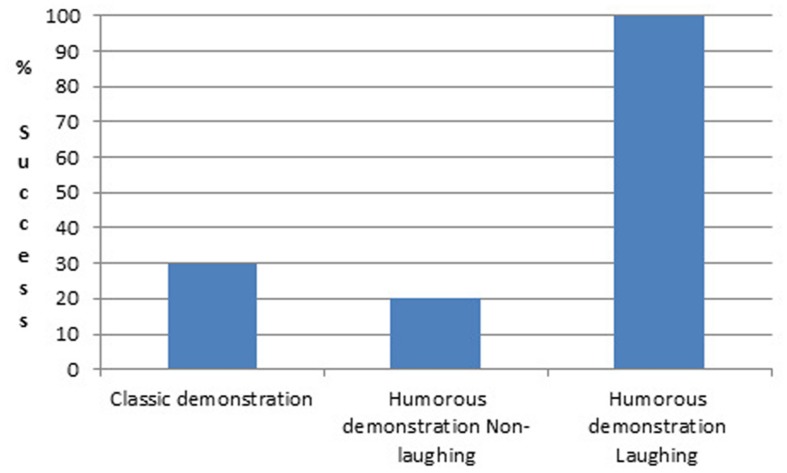
**Mean percentage of success as a function of group**.

Our results showed that the percentage of infants who successfully retrieved the toy using the tool is 30% in the Classic demonstration group, 20% in the Humorous demonstration/infants not laughing group, and 100% in the Humorous demonstration/infants laughing group, with these differences being statistically significant. The 30% success in the Classic demonstration group is close to the value found at 18 months in the spatial gap condition in Rat-Fischer et al.’s (2012) cross-sectional study. Interestingly, the laughing infants did not completely mimic the experimenter since only three of them threw the toy on the floor after retrieving it. All seven others kept the toy to play with. Thus, what was impressive is the way all laughing infants were able to make use of the demonstration to understand the usefulness of the tool in bringing the toy closer (see **Figure 7**).

Note that to be sure that success was due to the effect of laughing, and not due to differences in attention between the two groups, we checked and confirmed that the non-laughing infants looked at the experimenter during demonstrations as much as the laughing infants.

Two interpretations seem possible to explain these results. A first interpretation could be that the infants who were able to appreciate humor were more advanced in their social referencing or cognitive abilities. However, a suggestion that this is not correct comes from our observation that even the gazes of non-laughing infants were directed at the experimenter after she threw the toy on the floor, indicating that even non-laughing infants perceived the incongruity of the situation though it did not make them laugh. Nevertheless, a possible difference in social skills between laughing and non-laughing infants still needs to be explored. The second hypothesis is that of the role of positive emotions on learning, for example through endorphin release, known to facilitate cognitive flexibility (Ashby et al., 1999). This would fit with the observation we made in another study, showing that when infants are put in a positive emotional state, for instance when the experimenter mimics the infant’s action before the tool-use task, infants achieve higher level of success than when the test follows a more neutral pre-session (Somogyi and Esseily, 2014).

## Discussion

The goal of this article was to explore observational learning as a mechanism for learning tool use in the second year of life. We reviewed four published studies from our laboratory in which the demonstration was varied. In these studies there were four different conditions of demonstration: classic; showing intention prior to demonstration; repeated implicit demonstrations; and humorous demonstration. In addition, in the implicit demonstration study we opposed the benefit of implicit observational learning to that of manual familiarization without demonstration. The infants were tested at 16–18-months of age. In this age range, most infants who had not been first familiarized with the tool failed at the task (Rat-Fischer et al., 2012; Fagard et al., 2014).

When the demonstration was **classic**, i.e., when an adult explicitly showed the infant how to use the tool to retrieve the toy, there was no benefit from the demonstration at 16 months. Demonstration led to some successes starting at 18 months of age. This is late, compared with the success of 12- to 15-month-olds at other means-end tasks following demonstration (Provasi et al., 2001; Buttelmann et al., 2008; Esseily et al., 2010).

When the demonstration was preceded by a gesture toward the toy (**Prior intention**), thus indicating to the infant that the experimenter wanted to get the toy but could not grasp it directly, the effectiveness of a demonstration significantly increased: infants tried harder to retrieve the toy with the tool, even if they often did not fully succeed. Understanding the intention of an agent emerges around the first year of life (Bellagamba and Tomasello, 1999), especially when social cues are given (Carpenter et al., 1998). Our results are in line with studies showing that infants succeed more after demonstration if they have been informed about the experimenter’s intention (Carpenter et al., 2002; Southgate et al., 2009).

We then investigated whether the number of demonstrations could be an issue. We wanted to simulate a situation comparable to that of the spoon, where over an extended period of time, infants have many opportunities to watch people around them use a spoon, but without this observation being an explicit teaching situation. Thus in our experiment parents pushed the toys toward the infants using the tool, and did this without commenting, as if it was a natural thing to push a toy toward the infant using a rake (**Implicit repeated demonstrations**). In the same study, we contrasted this implicit visual training (the infants never had the opportunity to touch the tool) with a condition of manual familiarization without demonstration. The results showed a significantly greater benefit from visual familiarization compared to manual familiarization. In the test session not only did the Visual familiarization infants succeed spontaneously more often than the Manual familiarization infants, but among those infants who failed spontaneously, only those in the Visual familiarization group succeeded after demonstration.

Results from this third experiment show that 16-month-old infants rely more on observation than on their own motor experience when learning a new task that is relatively complex for their age. This conclusion extends the results of earlier studies involving older age groups (Hopper et al., 2010; Whiten and Flynn, 2010; Beck et al., 2011). This does not mean that manipulation is unimportant. Instead it is likely that observational learning and manipulation play a different role depending on the stage of learning. In our tool-use task, there are two factors of difficulty: first children must understand the affordance of the rake; once they know that they can use the rake to retrieve the toy, they must learn how to manipulate it in order to succeed. It was clear in our observations of the first behaviors of the children that they did not understand the affordance of the rake: they discarded it, or played with it after begging for the toy and without pointing the rake toward the toy. Once they tried to use the rake to retrieve the toy, the first such trials were unsuccessful because they did not know very well how to manipulate the rake, but success came rather quickly and within the same session. Observational learning seems more efficient than manipulation for discovering a complex affordance such as for an unfamiliar tool to retrieve an out-of-reach object, which requires bridging the gap between tool and toy before pulling back the toy with the tool. In turn, practice is important for refining the manual skill allowing the task to be done.

In the last study we checked the effect of laughing on observational learning (**Humorous demonstration**). We compared the effect of a classic demonstration with that of a humorous demonstration where the experimenter threw the toy on the floor after retrieving it with the tool. Only one third of the infants laughed, but 100% of the laughing infants fully succeeded after the demonstration, whereas there was a significantly lower percentage of success among the non-laughing infants and the infants in the classic demonstration condition. One important finding was that laughing infants’ success did not involve systematic mimicry of the adult’s action: the laughing infants clearly learned the affordance of the tool rather than a specific action of throwing the toy on the floor.

To summarize, this series of studies suggests that there might be several reasons why infants younger than 18 months do not learn to use a tool from a classic demonstration. First, not understanding the goal of the demonstrator may keep them from making sense of what they observe. Second, two demonstrations may not be enough to learn a complex affordance such as that of a tool: repeated demonstrations over an extended time period (weeks) may be necessary. Third, explicit teaching may not be the best way to help infants learn how to use a new tool. Fourth, an unexpected outcome leading to a shared positive emotional state is extremely effective in favoring learning from a demonstration. Another important conclusion from these studies is that, at least in the case of the rake, whose affordances may not be known to a child under 2 years of age, observational learning may be more important than manual practice in discovering new functions.

These findings share common interpretations with the studies on the factors influencing infants’ abilities to imitate mentioned in the introduction (opacity of the means, of the goal, of the model’s intention, irrationality of the means, etc.). Two interpretations can be proposed to explain them. One is that there is a cognitive load in understanding the demonstration of the model: the infant may not know what to attend to, what part of the action is important for success, when to pay attention, what is the affordance of the rake, etc. When the infant is shown the model’s intention before demonstration, when the goal is made clearer, when more demonstrations are provided, then the child better understands the affordance of the rake and how he or she should proceed to act like the model. Another, non-exclusive, interpretation is that learning is always based on social cues and interactions: not knowing how to reach a goal may be stressful for the young child, just as it is for adults, and the social cues given to the learner, and more generally the social context of the demonstration, may make a huge difference in the efficacy of the modeling of the action; this might explain why when there is no implicit pressure (such as “do like me”), or when the unexpected outcome puts the child in a positive emotional state, children are more likely to understand the means used by the model to retrieve the toy.

In conclusion, observational learning of complex new tasks in young children (as opposed to imitation of simpler tasks) is a somewhat neglected topic in developmental psychology. This review of studies we have done in our laboratory suggests that observational learning may be an important factor to consider in understanding the acquisition of tool use, in addition to more traditionally studied factors such as perceptual constraints, exploration and trial and error. Our review suggests that further work on observational learning should profitably include work on how a child interprets a demonstrator’s intentions, and on how implicit observation of non-teaching situations over extended time influences learning. Such studies may have pedagogical implications as concerns teaching new skills to very young infants.

## Author Contributions

JF and JO have co-supervised most of the experiments presented in this article. They wrote the article. They also conducted the longitudinal study presented first. LR-F is the main investigator of the first cross-sectional experiment (Classic demonstration), and the co-investigator of the other experiments presented in this article. She wrote the article where she is first author and participated to the other articles. RE is the co-main investigator with LR-F of the experiments “Intention prior to demonstration” and “Humorous demonstration,” and she wrote the articles where she is first author. ES is the main investigator of the experiment “Implicit repeated demonstration” and she has written the corresponding article.

## Conflict of Interest Statement

The authors declare that the research was conducted in the absence of any commercial or financial relationships that could be construed as a potential conflict of interest.

## References

[B1] Abravanel E. Levan Goldschmidt E. Stevenson M. B. (1976). Action imitation: the early phase of infancy. *Child Dev.* 47 1032–1044. 10.2307/1128440 1001087

[B2] Ashby F. G. Isen A. M. Turken A. U. (1999). A neuropsychological theory of positive affect and its influence on cognition. *Psychol. Rev.* 106 529–550. 10.1037/0033-295X.106.3.529 10467897

[B3] Ashford D. Davids K. Bennett S. J. (2007). Developmental effects influencing observational modelling: a meta-analysis. *J. Sports Sci.* 25 547–558. 10.1080/02640410600947025 17365541

[B4] Barrett T. M. Davis E. F. Needham A. (2007). Learning about tools in infancy. *Dev. Psychol.* 43 352–368. 10.1037/0012-1649.43.2.352 17352544

[B5] Bates E. Carlson-luden V. Bretherton I. (1980). Perceptual aspects of tool using in infancy. *Infant Behav. Dev.* 3 127–140. 10.1016/S0163-6383(80)80017-8

[B6] Beck B. B. (1980). *Animal Tool Behavior: The Use and Manufacture of Tools*. New York, NY: Garland Press.

[B7] Beck S. R. Apperly I. A. Chappell J. Guthrie C. Cutting N. (2011). Making tools isn’t child’s play. *Cognition* 119 301–306. 10.1016/j.cognition.2011.01.003 21315325

[B8] Bekkering H. Wohlschlager A. Gattis M. (2000). Imitation of gestures in children is goal-directed. *Q. J. Exp. Psychol. A* 53 153–164. 10.1080/713755872 10718068

[B9] Bellagamba F. Tomasello M. (1999). Re-enacting intended acts: comparing 12- and 18-month olds. *Infant Behav. Dev.* 22 277–282. 10.1016/S0163-6383(99)00002-8

[B10] Brown A. L. (1990). Domain-specific principles affect learning and transfer in children. *Cogn. Sci.* 14 107–133. 10.1016/0364-0213(90)90028-U

[B11] Buttelmann D. Carpenter M. Call J. Tomasello M. (2008). Rational tool use and tool choice in human infants and great apes. *Child Dev.* 79 609–626. 10.1111/j.1467-8624.2008.01146.x 18489416

[B12] Carpenter M. Akhtar N. Tomasello M. (1998). Fourteen through 18-month-old infants differentially imitate intentional and accidental actions. *Infant Behav. Dev.* 21 315–330. 10.1016/S0163-6383(98)90009-1

[B13] Carpenter M. Call J. Tomasello M. (2002). Understanding “prior intentions” enables two-year-olds to imitatively learn a complex task. *Child Dev.* 73 1431–1441. 10.1111/1467-8624.00481 12361310

[B14] Carpenter M. Call J. Tomasello M. (2005). Twelve- and 18-month-olds copy actions in terms of goals. *Dev. Sci.* 8 F13–F20. 10.1111/j.1467-7687.2004.00385.x 15647059

[B15] Chen Z. Siegler R. S. (2000). Across the great divide: bridging the gap between understanding of toddlers’ and older children’s thinking. *Monogr. Soc. Res. Child Dev.* 65 1–96. 12467094

[B16] Connolly K. Dalgleish M. (1989). The emergence of a tool-using skill in infancy. *Dev. Psychol.* 25 894–912. 10.1037/0012-1649.25.6.894

[B17] Elsner B. (2007). Infants’ imitation of goal-directed actions: the role of movements and action effects. *Acta Psychol. (Amst)* 124 44–59. 10.1016/j.actpsy.2006.09.006 17078915

[B18] Elsner B. Aschersleben G. (2003). Do I get what you get? Learning about the effects of self-performed and observed actions in infancy. *Conscious. Cogn.* 12 732–751. 10.1016/S1053-8100(03)00073-4 14656514

[B19] Elsner B. Hauf P. Aschersleben G. (2007). Imitating step by step: a detailed analysis of 9- to 15-month-olds’ reproduction of a three-step action sequence. *Infant Behav. Dev.* 30 325–335. 10.1016/j.infbeh.2006.10.001 17400048

[B20] Esseily R. Nadel J. Fagard J. (2010). Object retrieval through observational learning in 8- to 18-month-old infants. *Infant Behav. Dev.* 33 695–699. 10.1016/j.infbeh.2010.07.017 20708271

[B21] Esseily R. Rat-Fischer L. O’Regan K. Fagard J. (2013). Understanding the experimenter’s intention improves 16-month-olds’ observational learning of the use of a novel tool. *Cogn. Dev.* 28 1–9. 10.1016/j.cogdev.2012.10.001

[B22] Esseily R. Rat-Fischer L. Somogyi E. O’Regan K. J. Fagard J. (2015). Humour production may enhance observational learning of a new tool-use action in 18-month-old infants. *Cogn. Emot.* 10.1080/02699931.2015.1036840 [Epub ahead of print]. 25965997

[B23] Fagard J. (1998). “Changes in grasping skills and the emergence of bimanual coordination during the first year of life,” in *Clinics in Developmental Medicine: the Psychobiology of the Hand*, ed Connolly K. J. (Londress: MacKeith Press), 123–143.

[B24] Fagard J. Florean C. Petkovic M. Rat-Fischer L. Fattori P. O’Regan J. K. (2015). When do infants understand that they can obtain a desired part of a composite object by grasping another part? *Infant Behav. Dev.* 41 169–178. 10.1016/j.infbeh.2015.05.003 26275587

[B25] Fagard J. Lockman J. J. (2009). Change in imitation for object manipulation between 10 and 12 months of age. *Dev. Psychobiol.* 52 90–99. 10.1002/dev.20416 19937747

[B26] Fagard J. Rat-Fischer L. O’Regan J. K. (2014). The emergence of use of a rake-like tool a longitudinal study in human infants. *Front. Psychol.* 5:491 10.3389/fpsyg.2014.00491 PMC4033220 24904504

[B27] Gergely G. Bekkering H. Király I. (2002). Developmental psychology: rational imitation in preverbal infants. *Nature* 415:755. 10.1038/415755a 11845198

[B28] Gergely G. Csibra G. (2003). Teleological reasoning about actions: the naive theory of rational action. *Trends Cogn. Sci.* 7 287–292. 10.1016/S1364-6613(03)00128-1 12860186

[B29] Greif M. L. Needham A. (2011). “The development of human tool use in early life,” in *Tool use and Causal Cognition*, ed McCormack (Oxford: Oxford University Press), 51–68.

[B30] Hayes S. J. Ashford D. Bennett S. J. (2008). Goal-directed imitation: the means to an end. *Acta Psychol. (Amst)* 127 407–415. 10.1016/j.actpsy.2007.07.009 17880901

[B31] Hopper L. M. Flynn E. G. Wood L. A. N. Whiten A. (2010). Observational learning of tool use in children: investigating cultural spread through diffusion chains and learning mechanisms through ghost displays. *J. Exp. Child Psychol.* 106 82–97. 10.1016/j.jecp.2009.12.001 20064644

[B32] Huang C. T. Heyes C. Charman T. (2002). Infants’ behavioral reenactment of “failed attempts”: exploring the roles of emulation learning, stimulus enhancement, and understanding of intentions. *Dev. Psychol.* 38 840–855. 10.1037/0012-1649.38.5.840 12220059

[B33] Keen R. (2011). The development of problem solving in young children: a critical cognitive skill. *Annu. Rev. Psychol.* 62 1–21. 10.1146/annurev.psych.031809.130730 20822435

[B34] Lockman J. J. (2000). A perception–action perspective on tool use development. *Child Dev.* 71 137–144. 10.1111/1467-8624.00127 10836567

[B35] McGuigan N. Robertson S. (2015). The influence of peers on the tendency of 3-and 4-year-old children to over-imitate. *J. Exp. Child Psychol.* 136 42–54. 10.1016/j.jecp.2015.03.004 25897959

[B36] McGuigan N. Whiten A. Flynn E. Horner V. (2007). Imitation of causally opaque versus causally transparent tool use by 3- and 5-year-old children. *Cogn. Dev.* 22 353–364. 10.1016/j.jecp.2009.07.001

[B37] Meltzoff A. N. (1988). Infant imitation after a 1-week delay: long-term memory for novel acts and multiple stimuli. *Dev. Psychol.* 24 470–476. 10.1037/0012-1649.24.4.470 25147404 PMC4137879

[B38] Meltzoff A. N. (1995). Understanding the intentions of others - reenactment of intended acts by 18-month-old children. *Dev. Psychol.* 31 838–850. 10.1037/0012-1649.31.5.838 25147406 PMC4137788

[B39] Nagell K. Olguin R. S. Tomasello M. (1993). Processes of social learning in the tool use of chimpanzees (Pan troglodytes) and human children (Homo sapiens). *J. Comp. Psychol.* 107 174–186. 10.1037/0735-7036.107.2.174 8370271

[B40] Paulus M. Hunnius S. Vissers M. Bekkering H. (2011). Imitation in infancy: rational or motor resonance? *Child Dev.* 82 1047–1057. 10.1111/j.1467-8624.2011.01610.x 21679175

[B41] Piaget J. (1936). *La Naissance de l’Intelligence chez l’Enfant*. Neuchâtel: Delachaux et Niestlé.

[B42] Poulson C. L. Nunes L. R. Warren S. F. (1989). Imitation in infancy: a critical review. *Adv. Child Dev. Behav.* 22 271–298. 10.1016/S0065-2407(08)60417-6 2688378

[B43] Provasi J. Dubon C. D. Bloch H. (2001). Do 9- and 12-month-olds learn means-ends relation by observing? *Infant Behav. Dev.* 24 195–213. 10.1016/S0163-6383(01)00072-8

[B44] Rat-Fischer L. O’Regan J. K. Fagard J. (2012). The emergence of tool use during the second year of life. *J. Exp. Child Psychol.* 113 440–446. 10.1016/j.jecp.2012.06.001 22789968

[B45] Smitsman A. W. Cox R. F. A. (2008). Perseveration in tool use: a window for understanding the dynamics of the action-selection process. *Infancy* 13 249–269. 10.1080/15250000802004379

[B46] Somogyi E. Ara C. Gianni E. Rat-Fischer L. Fattori P. O’Regan J. K. (2015). The roles of observation and manipulation in learning to use a tool. *Cogn. Dev.* 35 186–200. 10.1037/a0019296

[B47] Somogyi E. Esseily R. (2014). Mimicry enhances observational learning in 16-Month-Old infants. *PLoS ONE* 9:e113695 10.1371/journal.pone.0113695 PMC4262202 25493561

[B48] Southgate V. Chevallier C. Csibra G. (2009). Sensitivity to communicative relevance tells young children what to imitate. *Dev. Sci.* 12 1013–1019. 10.1111/j.1467-7687.2009.00861.x 19840055

[B49] van Leeuwen L. Smitsman A. van Leeuwen C. (1994). Affordances, perceptual complexity, and the development of tool use. *J. Exp. Psychol. Hum. Percept. Perform.* 20 174–191. 10.1037/0096-1523.20.1.174 8133220

[B50] Want S. C. Harris P. L. (2001). Learning from other people’s mistakes: causal understanding in learning to use a tool. *Child Dev.* 72 431–443. 10.1111/1467-8624.00288 11333076

[B51] Whiten A. Flynn E. (2010). The transmission and evolution of experimental microcultures in groups of young children. *Dev. Psychol.* 46 1694–1709. 10.1037/a0020786 20822212

[B52] Whiten A. McGuigan N. Marshall-Pescini S. Hopper L. M. (2009). “Emulation, imitation, over-imitation and the scope of culture for child and chimpanzee.” *Philos*. *Trans. R. Soc. Lond. B Biol. Sci.* 364 2417–2428. 10.1098/rstb.2009.0069 PMC2865074 19620112

[B53] Willatts P. (1999). Development of means-end behavior in young infants: pulling a support to retrieve a distant object. *Dev. Psychol.* 35 651–667. 10.1037/0012-1649.35.3.651 10380857

[B54] Williamson R. A. Markman E. M. (2006). Precision of imitation as a function of preschoolers’ understanding of the goal of the demonstration. *Dev. Psychol.* 42 723–731. 10.1037/0012-1649.42.4.723 16802904

[B55] Zmyj N. Daum M. M. Aschersleben G. (2009). The development of rational imitation in 9-and 12-month-old infants. *Infancy* 14 131–141. 10.1080/15250000802569884 32693469

